# Simulated Wildfire Smoke Significantly Alters Sperm DNA Methylation Patterns in a Murine Model

**DOI:** 10.3390/toxics9090199

**Published:** 2021-08-27

**Authors:** Adam Schuller, Chiara Bellini, Timothy G. Jenkins, Matthew Eden, Jacqueline Matz, Jessica Oakes, Luke Montrose

**Affiliations:** 1 Department of Public Health and Population Science, Boise State University, Boise, ID 83725, USA; adamschuller@u.boisestate.edu; 2 Department of Bioengineering, Northeastern University, Boston, MA 02115, USA; c.bellini@northeastern.edu (C.B.); eden.m@northeastern.edu (M.E.); matz.ja@northeastern.edu (J.M.); j.oakes@northeastern.edu (J.O.); 3 Department of Cell Biology and Physiology, Brigham Young University, Provo, UT 84602, USA; tim_jenkins@byu.edu

**Keywords:** air pollution, particulate matter, sperm, male reproduction, epigenetics

## Abstract

Wildfires are now a common feature of the western US, increasing in both intensity and number of acres burned over the last three decades. The effects of this changing wildfire and smoke landscape are a critical public and occupational health issue. While respiratory morbidity due to smoke exposure is a priority, evaluating the molecular underpinnings that explain recent extrapulmonary observations is necessary. Here, we use an Apoe^−/−^ mouse model to investigate the epigenetic impact of paternal exposure to simulated wildfire smoke. We demonstrate that 40 days of exposure to smoke from Douglas fir needles induces sperm DNA methylation changes in adult mice. DNA methylation was measured by reduced representation bisulfite sequencing and varied significantly in 3353 differentially methylated regions, which were subsequently annotated to 2117 genes. The differentially methylated regions were broadly distributed across the mouse genome, but the vast majority (nearly 80%) were hypermethylated. Pathway analyses, using gene-derived and differentially methylated region-derived gene ontology terms, point to a number of developmental processes that may warrant future investigation. Overall, this study of simulated wildfire smoke exposure suggests paternal reproductive risks are possible with prolonged exposure.

## 1. Introduction

Wildfire smoke exposure poses a major risk to human health in both a community and occupational setting [1]. Wildfire activity is increasing concurrent with climate change and smoke-generating events are becoming more frequent and longer in duration [2]. Wildfire smoke is comprised of thousands of chemicals which vary widely based on the conditions surrounding combustion [3]. Particulate matter (PM) air pollution makes up the largest weight by volume of wildfire smoke and is often used as a proxy for exposure intensity [4]. PM with an aerodynamic diameter of 2.5 μm or smaller poses the greatest risk to human health due to its ability to be inhaled, delivered to the deep lung, and to penetrate the air/blood barrier [5]. In addition to respiratory morbidity, increased exposure to woodsmoke PM has been correlated with extra-pulmonary health effects, including impacts to the heart, brain, and reproductive organs [6,7,8].The portion of total US PM made up by wildfire smoke PM is nearing 50% for some regions and this is concerning given recent reports that PM from wildfires may be more toxic compared to other sources [9,10]. Thus, characterization of wildfire smoke PM effects, independent from other ambient sources, is necessary.

In an effort to study extrapulmonary tissue-specific effects related to wildfire smoke PM exposure, epigenetic modifications are of interest. Epigenetic alterations are known to occur in response to environmental exposures, including woodsmoke [11]. These exposure-induced changes may represent useful biomarkers for direct PM exposure or indirect exposure via signaling molecules, such as inflammatory cytokines. Alternatively, epigenetic aberrancies may influence disease risk in a causal manner. DNA methylation—the most well-studied epigenetic alteration—is a chemical modification of a cytosine base in a cytosine-phosphate-guanine dinucleotide (CpG) via addition of a methyl group at the 5-carbon position. Perinatal DNA methylation modifications, established via a process of gene imprinting, are particularly impactful as they can be retained during cell division, affect other types of epigenetic patterns, and regulate gene transcription [12]. Aberrant DNA methylation of sperm-imprinted genes has been associated with male infertility [13]. More recently, paternal biomass combustion exposure was correlated with abnormal offspring behavior, which was speculated to be the result of inherited differential DNA methylation via sperm [14]. Notably, in the mammalian genome, CpGs are not distributed evenly across the genome [15]. Here, we present data using reduced-representation bisulfite sequencing (RRBS), which measures DNA methylation patterns at genomic sites, such as promoter regions, that are enriched for CpGs. Our data indicate, for the first time, differential DNA methylation patterns in the sperm of adult Apoe^−/−^ mice exposed to a laboratory-scale model of wildfire smoke PM relative to fresh air controls.

## 2. Materials and Methods

### 2.1. Animals

#### 2.1.1. Ethics Statement

All animal experiments complied with NIH guidelines and were performed under protocols that were approved by Northeastern University’s Institutional Animal Care and Use Committee (IACUC, protocol #20-0731R). The 8-week-old male Apoe^−/−^ mice on a C57BL/6 background were all obtained from The Jackson Laboratory (Bar Harbor, ME, USA). Apoe^−/−^ mice were chosen as they are a well characterized co-morbidity model, which may be amplified with chronic exposure to smoke [16]. Mice were housed in clear plastic cages in groups of up to 5, with ad libitum food and water. All cages were housed in a vivarium which was temperature-controlled and on a 12 h light/dark cycle.

#### 2.1.2. Experimental Design

The 8-week-old Apoe^−/−^ mice were randomly assigned to one of two groups: smoke-exposed mice (*n* = 10) or fresh air control mice (*n* = 10). Mice were exposed for 2 h per day, 5 days per week for a total of 40 days. All mice were sacrificed approximately 48 h following the final day of exposure. Sperm DNA methylation analysis was performed by RRBS to investigate the impact of wildfire smoke exposure on reproductive tissue.

#### 2.1.3. Douglas Fir Smoke Exposure

Simulated wildfire smoke was generated through a quartz-tube and ring furnace which was mounted on a linear actuator, based on German apparatus DIN 53436 [17]. Dried Douglas fir tree needles, sourced from Western Montana, were loaded onto the quartz-tube to be used to generate wildfire smoke. Controlled smoldering combustion was achieved by setting the quartz-tube furnace to 450 °C and the furnace travel speed to 20 mm/min. Desired smoke concentrations were set to 20 mg/m^3^, as determined by equating surface-area normalized deposited particulate mass between mouse and man, assuming approximately 15 years of wildland firefighting service. This was achieved by diluting the wildfire smoke with filtered lab air. Mice were whole-body exposed in a custom-built 3-D printed exposure chamber, which was designed and experimentally validated to evenly distribute smoke.

### 2.2. Molecular Analyses

#### 2.2.1. Sperm Collection and DNA Extraction

Animal sacrifice and sperm collection were conducted at Northeastern University. Using a dissecting scope, sperm was collected from the *cauda epididymis* and *vas deferens* immediately after euthanasia by scoring the tissue along the length of the tubules with fine forceps and aspiration of sperm mass via an insulin syringe. Tissues were placed in a Petri dish in HBSS. Sperm samples were then transferred with buffer into microcentrifuge tubes and snap frozen on liquid nitrogen. Subsequently, samples were shipped on dry ice to Boise State University where they were stored at −80 °C. Samples were thawed and subjected to a stringent somatic cell lysis protocol to ensure a pure population of sperm. Briefly, cells were first washed in 10 mL of PBS the centrifuged to remove the supernatant. Next, 10 mL of somatic cell lysis buffer (0.5% Triton-x100 and 0.1% SDS) were added to each sample, mixed and incubated over night at 4C. The following day, samples were centrifuged and the supernatant discarded. Samples were then washed one final time with PBS and the supernatant was decanted. Following somatic cell lysis, DNA was extracted using a sperm-specific modification to the Qiagen DNeasy kit (Hilden, Germany). The main modification was to precede the DNeasy instructions with the addition of a sperm cell lysis buffer which includes 20 mM TrisCl (pH 8.0), 20 mM EDTA, 200 mM NaCl, 80 mM DTT, 4% SDS, and 250 μg/mL Proteinase K. Following an overnight incubation at 56C, the sample was processed according to DNeasy extraction kits manufacturer’s instructions for cells. Extracted DNA samples were stored at −20 °C until shipment to Novogene.

#### 2.2.2. RRBS Library Construction

Initial concentrations of sperm DNA were completed at Boise State University and the samples were then shipped to Novogene on dry ice per the company’s instructions. RRBS sample preparation, sequencing and initial bioinformatic analysis was conducted by Novogene (Beijing, China). Sperm DNA concentration and quantification were confirmed by quibit and agarose gel electrophoresis, respectively. All 20 sperm DNA samples were classified as acceptable to proceed to library construction. Lambda DNA was spiked into each sample to monitor conversion efficiency. Digestion was completed with the enzyme MspI followed by repair with dA-tails and ligation to sequencing adaptors (5′ Adapter: 5′-AGATCGGAAGAGCGTCGTGTAGGGAAAGAGTGT-3′, 3′ Adapter: 5′-GATCGGAAGAGCACACGTCTGAACTCCAGTCAC-3′). DNA fragments were size selected with insertion lengths ranging from 40 bp to 220 bp using gel cutting [18]. Then, fragments were bisulfite treated with an EZ DNA Methylation Gold Kit (Zymo Research, Irvine, CA, USA), where unmethylated cytosines are changed to uracil and methylated cytosines remain unchanged. The final DNA library was obtained by PCR amplification following a standard procedure. Library preparation was completed with Accel-NGS^®^ Methyl-Seq DNA Library Kit. Library size was detected with an Agilent 2100 and adequate concentration was confirmed by q-PCR. Sequencing was performed on the HiSeq/NovaSeq (Illumina, San Diego, CA, USA) platform.

#### 2.2.3. Bioinformatics Analyses

The original images obtained by the sequencing platform are transformed by CASAVA base calling and saved in fastq format. FastQC version 0.11.5 was used to get basic statistics and ensure quality. The Illumina NGS data tool, Trimmomatic version 0.36, was then used to filter out contaminated adapter sequence and low-quality reads. Bismark software version 0.16.3 was used to align bisulfite treated reads to the mm10 reference mouse genome [19]. The threshold for accurate methylation sites was a sequencing depth greater than or equal to 5× and a q-value less than or equal to 0.01 [20,21]. Differentially methylated regions (DMRs) were identified using the open source R package dispersion shrinkage for sequencing data (DSS version 2.12.0) with the following parameters: delta = 0, *p* value threshold = 1 × 10^−5^, minimum length = 50 base pairs, and minimum CpGs = 3 [22]. The significance of a DMR was weighted by the ‘areastat’, which is the sum of the t-statistic values in each DMR.

#### 2.2.4. Gene Ontology

Gene ontology (GO) term analysis is a standard method of classifying gene function based on shared relationships between specific elements of the genome, which can be individual loci, regions, or genes [23]. For this study we performed two types of GO term analyses. First, as part of the RRBS pipeline (conducted by Novogene), DMRs were annotated to genes, and those genes were then used in a GO term analysis. Second, we performed GO term analysis on the full set of DMRs, which included coding and non-coding regions, using the GREAT [24] online tool version 4.0.4.

## 3. Results

### 3.1. Overview of DNA Methylation Characteristics

**Figure 1:**
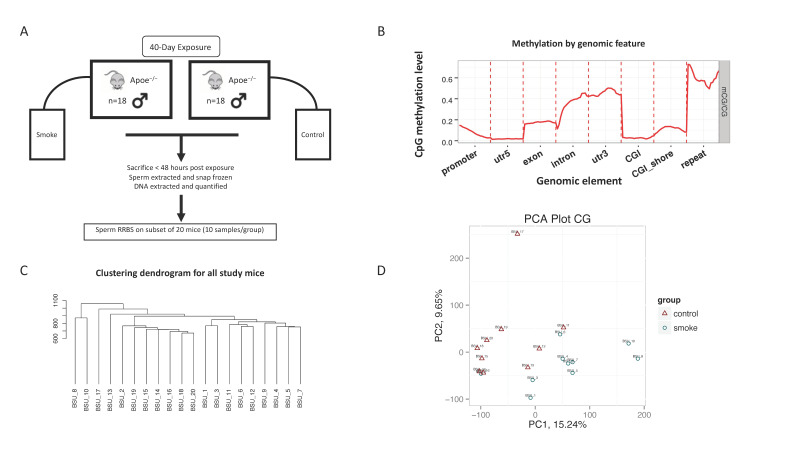
Schematic of study design and DNA methylation characteristics. (**A**) Study design showing exposure group, study duration and health outcome of interest. (**B**) An example of the average methylation level distribution in genomic functional regions for one of the samples is shown to demonstrate general agreement with our understanding of methylation profiles (i.e., promoter methylation is relatively low, while repeat elements are highly methylated). (**C**) The dendrogram shows the hierarchical clustering of the study mice. (**D**) PCA plot for principal components 1 and 2. Panels C and D demonstrate the relationship between samples, which is mostly dichotomous by exposure group, where IDs “BSU 1-10” are exposed and “BSU 11-20” are control.

RRBS was performed on 20 mouse sperm DNA samples. The mapping rate was 69.9% and the bisulfite treatment efficiency was 98.6% on average, with a range from 98.1% to 98.8%. The percent of bases with a minimum of 5× coverage was 26.1% and 10x coverage was 23.7%. Mean coverage depth for cytosines in a CpG context was 27.3%. The overall methylation profiles are consistent with expected results from quality samples, with promoters mostly unmethylated and repeat elements mostly methylated (Figure 1B). Figure 1C,D show the results of unsupervised computational analysis and demonstrate that the sperm DNA methylation profiles are clustered by exposure group. The average (range) of DNA methylation at CpGs captured by RRBS in this mouse study was 1.49% (1.19–1.68%).

### 3.2. Simulated Wildfire Smoke-Induced Changes in Sperm DNA Methylation

**Figure 2:**
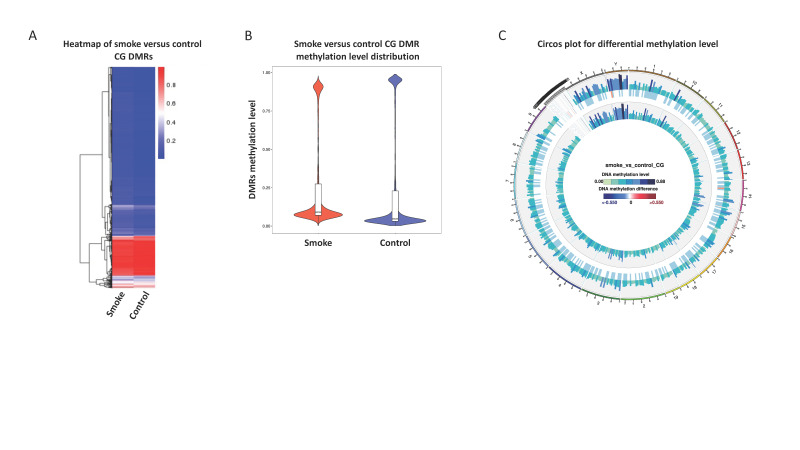
Differential methylation of smoke-exposed versus non-exposed mice. (**A**) Heatmap of DMRs separated by exposure group and representing all 20 animals. (**B**) Violin plot showing average methylation level for DMRs separated by exposure group. (**C**) The Circos plot reflects the difference of the combined methylation levels of the exposure groups. Methylation of the smoke-exposed mice are around the outside and methylation of the non-exposed mice are around the inside. In between is the methylation difference between exposure group with blue denoting that the smoke-exposed mice have a more methylated profile and red denoting a less methylated profile.

We explored the effects of 40 day wildfire smoke exposure on mouse sperm DNA. Using RRBS and a regional analysis, 3353 DMRs were identified when comparing the smoke-exposed to the control mice, with 703 (21%) being hypomethylated and 2650 (79%) being hypermethylated. Among these smoke-induced DMRs, 2117 genes were found to be associated with the DMRs. Figure 2A shows the DMRs of the exposed and non-exposed mice side by side. While both groups have heat maps reflecting the profile of a sperm cell, there is a subtle visual distinction with the blue and red tracks of the smoke-exposed group being a lighter shade. Similarly, comparing the average methylation (Figure 2B) of the DMRs demonstrates that the bimodal distribution of sperm DNA methylation pattern is subtly shifted toward the center (i.e., genes that are normally hyper- or hypomethylated are less so in the smoke group relative to the control). Note that the RRBS platform is enhanced for regions that may be functionally relevant for gene expression, which are more often hypomethylated. This explains why the lower distribution of the violin plot is larger than the upper. The DMRs are distributed broadly throughout the genome but are not evenly split between hyper- and hypomethylated shifts, evidenced by shifts toward more methylation seen in the Circos plot (Figure 2C).

### 3.3. GO Term Analysis for DMR-Annotated Genes

**Figure 3:**
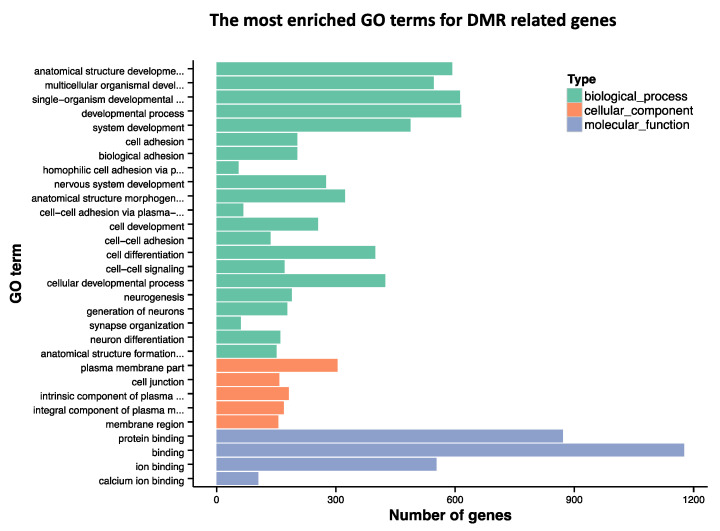
GO term analysis for genes annotated to DMRs. The bar graph shows the GO terms categorized by biological process, cellular component and molecular function. The GO terms represent gene functions that are related to groups of genes which were annotated to DMRs in our study.

GO analysis was conducted on the 2117 annotated genes and identified multiple gene classes that were enriched in smoke-exposed mice compared to control (Figure 3). This pathways analysis identified 21 biological processes, 5 cellular components, and 4 molecular functions with an adjusted *p* < 0.05.

### 3.4. GO Term Analysis for DMRs

GO analysis was conducted on the 3353 DMRs, including both coding and non-coding regions, and identified multiple gene classes that were enriched in smoke-exposed mice compare to non-exposed (Supplementary Table S1). This pathways analysis identified 24 biological processes, 5 cellular components, and 2 molecular functions with an adjusted *p* < 0.05.

## 4. Discussion

In this work, we studied the effect of simulated wildfire smoke generated in a laboratory setting on sperm DNA methylation. We found a robust set of DMRs in mouse sperm DNA following exposure to smoke at levels and duration relevant to human occupational exposure (e.g., approximately 15 years of wildland firefighting service) compared to fresh-air controls. Epigenetic modifications following environmental exposure events are emerging as potential mechanistic drivers of adverse human health outcomes that remain idiopathic in origin. DNA methylation remains the most stable epigenetic modification, both in terms of mitotic heritability and in post-mortem stability [25,26]. Appropriate DNA methylation is paramount for normal gene regulation [27], mammalian development [28], and disease risk [29]. The GO terms associated with the genes (Figure 3) and DMRs (Supplementary Table S1) identified in this study include many developmental processes. While this is the first time that sperm DNA methylation patterns have been examined following wildfire smoke exposure, our results are consistent with GO term results from an existing study assessing tobacco and cannabis smoke exposure, including gene networks important for neuronal development and neuronal processes [30]. Notably, these sources of air pollution PM likely differ in toxic potential, which the authors posit warrants the continued assessment of wildfire smoke separately from the existing works. The relationship between wildfire smoke and brain health has been recently reviewed by our group [7] and others [31]. The impact of germline epigenetic changes on offspring central nervous system disease risk also warrants future study.

The toxicity of combustion-related PM varies based on the fuel source and combustion conditions. Studies on wildfire smoke combustion suggest that the toxic potentials of flaming versus smoldering fires differ due in part to the unique chemical profile produced under both conditions [32]. It will be important for future experiments to clearly distinguish a range of laboratory combustion conditions in addition the effects of smoldering smoke, which could include flaming smoke and flaming/smoldering mixtures as well as individual and mixtures of wildfire-relevant fuel sources. Similar to our results demonstrating differential sperm DNA methylation changes following exposure to smoldering Douglas fir needles, others have shown epigenetic changes in response to additional combustion sources such as cigarette [33,34] and cannabis smoke [35]. Different from the results of the current study, Jenkins et al. previously has shown that cigarette smoke [33] as well as age [36] is associated with a trend toward CpG level and regional hypomethylation in human sperm, whereas the wildfire smoke-induced changes presented here were largely in a hypermethylated direction. Global hypermethylation has been shown to occur in mouse sperm following 10 weeks of exposure to urban ambient PM exposure [37]. However, it is difficult to compare DNA methylation profiles across studies which used varying analytical techniques and scale of analysis (some targeting individual CpGs, genomic regions, or global analysis) as these approaches can differ in the level of bias for CpG richness and function.

DNA methylation is indicative of overall male reproductive health as it has been associated with abnormal sperm counts and motility, and may also act as a biomarker of multigenerational disease risk [38,39]. DNA methylation patterns are reprogrammed at specific times during early development, first with the genesis of primordial germ cells in a newly developing embryo and again immediately following fertilization [40]. These waves of erasure, which include the paternal germ-line methylome may remove exposure-induced differential DNA methylation. However, the literature remains mixed on the possibility of exposure-induced intergenerational and/or transgenerational inheritance of epigenetic marks via the male germ line [41]. Here, intergenerational inheritance is defined as the passing of exposure-induced epigenetic information from one generation to the next, whereas transgenerational inheritance is defined as the passing of exposure-induced epigenetic information between generations in the absence of continued exposure [42]. Transgenerational inheritance has been reported in plants, rodents, and humans [43,44,45]. Recent work from Skinner et al. suggests that DDT exposure can induce alterations in multiple epigenetic patterns, including DNA methylation in a transgenerational fashion, but it is notable that the differential patterns were not consistent between generations [46]. At the same time, while some have demonstrated epigenetic transgenerational inheritance following exposure to endocrine disruptors (EDCs) [45], others have shown the paternal germline epigenetic alterations induced by similar EDC mixtures are corrected and do not impact the offspring [47]. The persistence of wildfire smoke-induced sperm DNA methylation and its ability to evade erasure are important questions that should be addressed in future animal and human studies.

## 5. Conclusions

Simulated wildfire smoke at human occupationally relevant levels is sufficient to alter the methylome of mouse sperm DNA. The overall implications of these findings for the health of humans are unclear. While intergenerational or transgenerational effects remain debatable, our findings of exposure-induced differential DNA methylation in mouse sperm are novel and important in the context of a growing body of literature characterizing biomarkers of exposure to wildfire smoke. The sensitivity of sperm DNA methylation patterns to wildfire smoke, tobacco smoke, cannabis smoke, and urban PM highlights the need to understand the mechanism of action, which may be shared between these exposures.

## Data Availability

RRBS data will be made publicly available at GEO (https://www.ncbi.nlm.nih.gov/geo/, accessed on 26 August 2021).

## Supplementary Materials

The following are available online at https://www.mdpi.com/article/10.3390/toxics9090199/s1. Table S1: GO term analysis for DMRs.
